# Cardiovascular risks and bleeding with non-vitamin K antagonist oral anticoagulant versus warfarin in patients with type 2 diabetes: a tapered matching cohort study

**DOI:** 10.1186/s12933-020-01152-y

**Published:** 2020-10-10

**Authors:** Dahai Yu, Zhanzheng Zhao, David Simmons

**Affiliations:** 1 Department of Nephrology, The First Affiliated Hospital, Zhengzhou University, Zhengzhou, 450052 China; 2 Primary Care Centre Versus Arthritis, School of Medicine, Keele University, Keele, ST5 5BG UK; 3 Macarthur Clinical School, School of Medicine, Western Sydney University, Locked Bag 1797, Campbelltown, Sydney, NSW 2751 Australia

**Keywords:** NOAC, Warfarin, Bleeding, Cardiovascular disease, Hospitalisation, Tapered matching, Non-vitamin K antagonist oral anticoagulants

## Abstract

**Background:**

We compared the risk of bleeding and cardiovascular disease (CVD) events between non-vitamin K antagonist oral anticoagulant (NOAC) and warfarin in people with type 2 diabetes (T2DM).

**Methods:**

862 Incident NOAC users and 626 incident warfarin users with T2DM were identified from within 40 UK general practice (1/4/2017–30/9/2018). Outcomes included incident hospitalisation for bleeding, CVD and re-hospitalisation for CVD within 12 months since first anticoagulant prescription, identified from linked hospitalisation data. A tapered matching method was applied to form comparison cohorts: coarsened exact matching restricted the comparison to areas of sufficient overlap in missingness and characteristics: (i) demographic characteristics; (ii) clinical measurements; (iii) prior bleeding and CVD history; (iv) prescriptions with bleeding; (v) anti-hypertensive treatment(s); (vi) anti-diabetes treatment(s). Entropy balancing sequentially balanced NOAC and warfarin users on their distribution of (i–vi). Weighted logistic regression modelling estimated outcome odds ratios (ORs), using entropy balancing weights from steps i–vi.

**Results:**

The 12-month ORs of bleeding with NOAC (n = 582) vs matched/balanced warfarin (n = 486) were 1.93 (95% confidence interval 0.97–3.84), 2.14 (1.03–4.44), 2.31 (1.10–4.85), 2.42 (1.14–5.14), 2.41 (1.12–5.18), and 2.51 (1.17–5.38) through steps i–vi. ORs for CVD re-hospitalisation was increased with NOAC treatment through steps i–vi: 2.21 (1.04–4.68), 2.13 (1.01–4.52), 2.47 (1.08–5.62), 2.46 (1.02–5.94), 2.51 (1.01–6.20), and 2.66 (1.02–6.94).

**Conclusions:**

Incident NOAC use among T2DM is associated with increased risk of bleeding hospitalisation and CVD re-hospitalisation compared with incident warfarin use. For T2DM, caution is required in prescribing NOACs as first anticoagulant treatment. Further large-scale replication studies in external datasets are warranted.

## Background

Anticoagulants are used for the prevention and treatment of venous thromboembolism and to reduce the risk of stroke in patients with either atrial fibrillation or after acute pulmonary embolism, deep vein thrombosis [1, 2]. Warfarin has been used for six decades but in the last 8 years its use has been gradually replaced by non-vitamin K antagonist oral anticoagulants (NOACs) including dabigatran, rivaroxaban, and apixaban. Unlike warfarin, these drugs have set doses and do not generally require regular international normalisation ratio blood test monitoring [3]. They also have faster onset and offset of action. There are, however, some concerns regarding the safety of NOACs with respect to bleeding because there is a limited choice of antidotes, experience with reversal agents is limited outside of trials and some remain expensive [4, 5].

Studies in the general population have reported potential further clinical benefits of NOACs over warfarin, for example, QResearch found potential for apixaban to be associated with a decreased risk of major bleeding events in patients with and without atrial fibrillation compared with warfarin; rivaroxaban is associated with a decreased risk of intracranial bleeding in patients without atrial fibrillation compared with warfarin; rivaroxaban and low dose apixaban are associated with an increased risk of all-cause mortality in patients with and without atrial fibrillation compared to warfarin [6]. However, few observational cohort studies have addressed short-term outcomes such as hospitalisation due to bleeding and cardiovascular disease (CVD), which are linked to significant health costs [7, 8]. Moreover, few studies have examined such associations in populations with type 2 diabetes, which is distinctively different from the general population, in terms of age, and comorbidities [9].

To compare the risks of major bleeding and CVD hospitalisation between incident NOAC and warfarin users with type 2 diabetes, we have used a recently introduced statistical approach: tapered multivariable matching [10, 11]. This allows us to examine the extent of the observed difference in these outcomes between NOAC and warfarin users, and more importantly to investigate how potential confounders relate to the risk differentiation. In tapered matching, we sequentially match NOAC user group to the warfarin user group with an increasingly comprehensive set of variables. As we incrementally match the NOAC user group and the warfarin user group, we can directly observe how the matched cohorts change both in terms of risk of outcome and in terms of unmatched covariables.

## Methods

### Data sources

Two UK primary care databases from two clinical commissioning groups (CCGs) in England were used for this study. All 21 practices in CCG-1 and 19 practices in CCG-2 practices were linked at the patient level to hospital admission data (Secondary User Service (SUS) data). We used READ codes to extract the information from general practices [12, 13] and ICD-10 (international classification of diseases, 10th revision) codes for outcomes extracted from SUS data (codes list is accessible by reasonable request via corresponding author) [14, 15]. Anonymised data were used in this study. The study was approved by South West—Exeter Research Ethics Committee (REC reference: 17/SW/0001).

The study period ran from 1 January 2017 to 30 September 2018. All patients with type 2 diabetes newly prescribed the oral anticoagulants warfarin, dabigatran, rivaroxaban, and apixaban, and aged from 18 to 99 years at the date of study entry, were eligible to be enrolled in this study. The entry date was defined as the date of the first prescription of any of the anticoagulant drugs. To facilitate a direct comparison between new users of NOACs against new users of warfarin, and to reduce the impact of indication bias, patients were excluded if they had any anticoagulant prescription in the prior 12 months before the entry date. To ensure the quality of data, patients were also excluded if they had less than 12 months of registration history before entry. International Normalized Ratio (INR) monitoring was considered a key component of warfarin management and as such included in the term “warfarin use”. INR information was not available in this study.

The outcome of interest included (i) incident hospitalisation due to CVD defined by the primary ICD-10 code within the 12 months following the first anticoagulant prescription; (ii) incident hospitalisation mainly due to bleeding events defined by the primary ICD-10 codes within the 12 months since the first anticoagulant prescription; (iii) re-hospitalisation mainly due to CVD (having ≥ 2 hospitalisations mainly due to CVD) defined by the primary ICD-10 codes within the 12 months since the first anticoagulant prescription. It is important to note that other conditions defined by non-primary ICD-10 codes could have contributed to the hospitalisation, but these have not been used in the definition of the outcomes.

Patients were followed from their first prescription of an anticoagulant until they experienced an outcome of interest or by the 12 months without experiencing any outcome of interest. Patients were excluded if they stopped or suspended treatment up to 30 days after the first anticoagulant prescription. Patients were excluded if they switched between NOAC and warfarin within the first 12 months.

### Statistical methods

#### Matching method

**Fig. 1:**
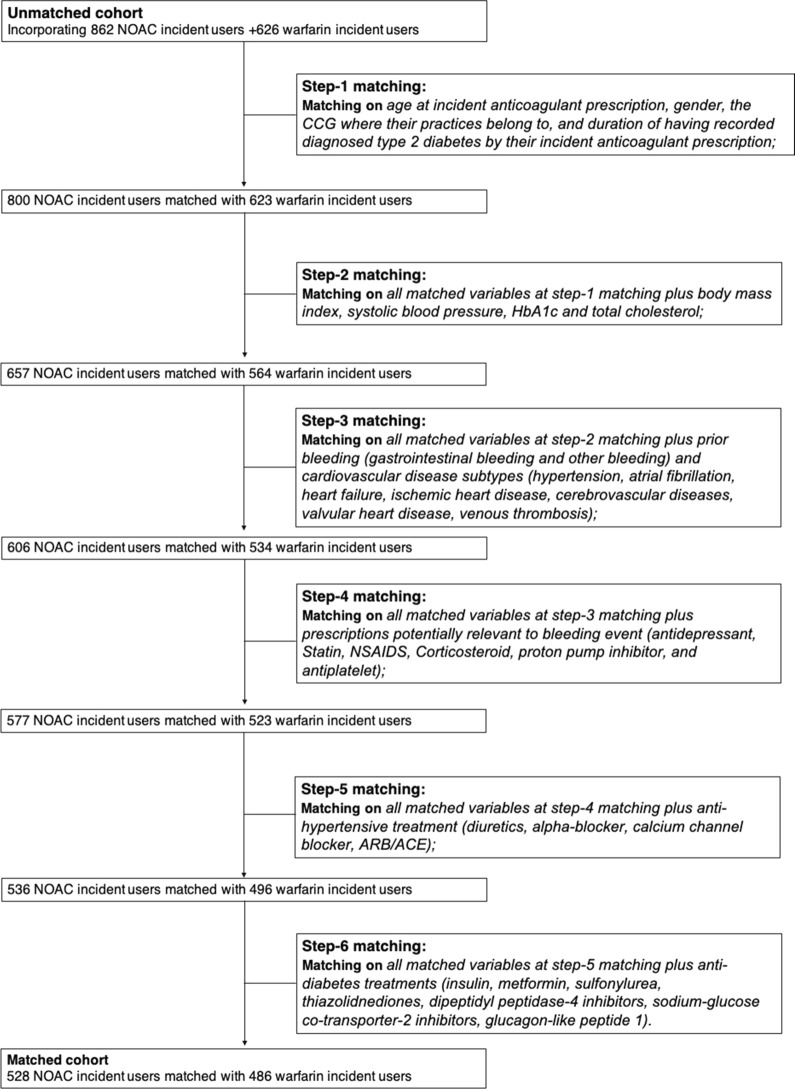
Workflow charts for matching process

This study used a tapered matching method to generate a series of matches for each comparison of NOAC and warfarin [16]. For the NOAC user group, we performed six matches that constructed sets of pairs of warfarin users as shown in Fig. 1. First, demographic factors match paired patients by their age at incident anticoagulant prescription, gender, the CCG associated with their practice, duration of recorded diagnosed type 2 diabetes by their incident anticoagulant prescription, and issue year of incident anticoagulant prescription. Second, the NOAC user group match controlled (warfarin user group) for all demographic factors and clinical measurements (body mass index, systolic blood pressure, HbA1c and total cholesterol). Third, the NOAC user group were matched with the warfarin user group for all variables in the first 2 matches as well as prior bleeding (gastrointestinal bleeding and other bleeding) and CVD subtypes (hypertension, atrial fibrillation, heart failure, ischemic heart disease, cerebrovascular diseases, valvular heart disease, venous thrombosis); Fourth, the NOAC user group were matched with the warfarin user group for all variables in the first 3 matches as well as prescriptions potentially relevant to bleeding event (antidepressant, Statin, NSAIDS, Corticosteroid, proton pump inhibitor for gastrointestinal disease, and antiplatelet). Fifth, the NOAC user group were matched with the warfarin user group for all variables in the first 4 matches as well as anti-hypertensive treatment (diuretics, alpha-blocker, calcium channel blocker, ARB/ACE). Sixth, the NOAC user group were matched with the warfarin user group for all variables in the first 5 matches as well as difference in anti-diabetes treatments (insulin, metformin, sulfonylurea, thiazolidinediones, dipeptidyl peptidase-4 inhibitors, sodium-glucose co-transporter-2 inhibitors, glucagon-like peptide 1). In each step of matching, we use coarsened exact matching (CEM) algorithm that is a monotonic imbalance reducing matching method, which means that the balance between the treated and control groups is chosen by *ex ante* user choice rather than discovered through the usual laborious process of “checking after the fact, tweaking the method, and repeatedly re-estimating” [17]. Patients both in the NOAC and warfarin user groups matched on step-6 were retained (Fig. 1).

Via CEM we restricted the comparison of NOAC and warfarin user groups to areas of common support, i.e. sufficient overlap between the two groups, on the above key factors in the six-steps, coarsened using the default Sturges measure of bin size [18]. After excluding patients (Fig. 1) who were off common support, we then used entropy balancing [19] to efficiently minimise differences in the distribution of matching variables between NOAC and warfarin user groups. Entropy balancing involves maximum entropy reweighting the matched sample in each matching step to key target moments (mean, variance and skewness). For continuous matching variables, all three moments should be met; for binary variables the only target moment is the mean as it is only sufficient to match higher moments (variance and skewness).

Weighted logistic regression, incorporating matching weights estimated from each matching step by entropy matching, was applied in each matching step. This provided an estimate of the association between NOAC use and risk of hospitalisation mainly due to bleeding and CVD, with warfarin as the reference group [19].

## Results

**Fig. 2:**
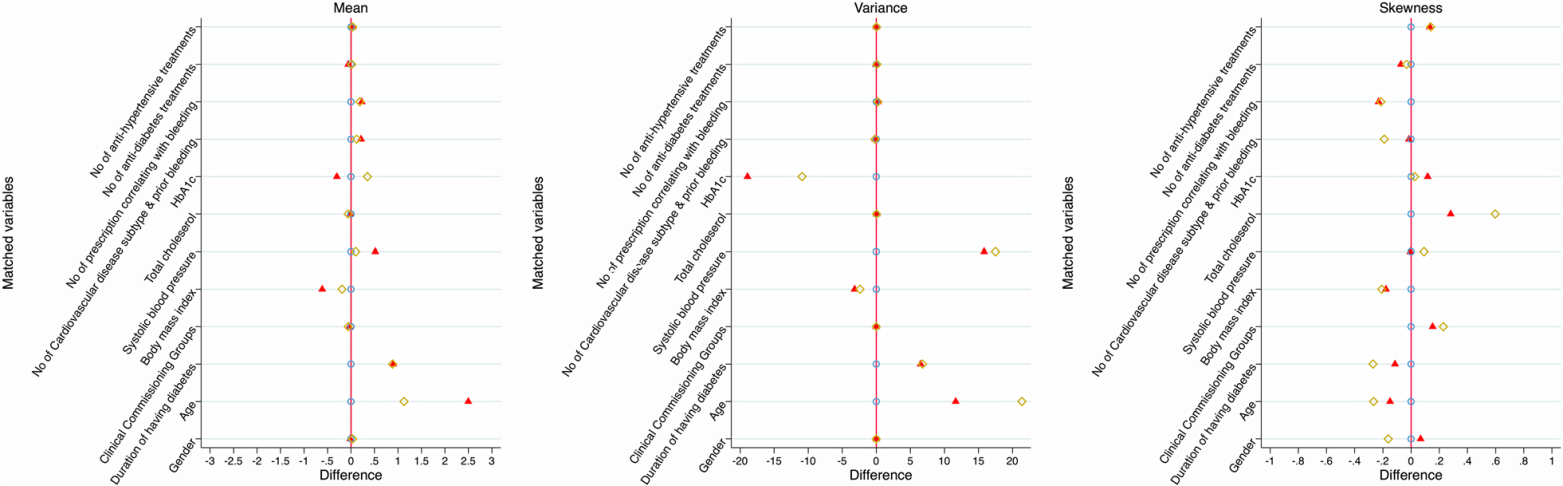
Distribution of difference of means, variance and skewness on matched variables in the unmatched and matched cohorts. Triangles indicate measurements from unmatched cohorts; diamonds indicate measurements from coarsened exact matching; circles indicate measurements from entropy matching cohorts

We identified 862 people with type 2 diabetes with incident NOAC prescription and 626 people with type 2 diabetes with incident warfarin prescription between 2017 and 2018 in the two CCGs. Table 1 and Fig. 2 demonstrate that the matched variables were quite different between NOAC users and warfarin users. For example, NOAC users have a lower HbA1c with fewer existing bleeding or CVD comorbidities comparing with warfarin users (Table 1). The distributions of outcomes in the unmatched cohorts and cohorts matched by coarsened exact matching are presented in Table 2.

**Table 1:** Baseline characteristics in the comparison cohorts

	Unmatched cohorts			Cohorts after coarsened exact matching		
	Incident NOAC users (N = 862)	Incident warfarin users (N = 626)	P-value for matching variables	Incident NOAC users (N = 582)	Incident warfarin users (N = 486)	P-value for matching variables
Clinical commission group-2, n (%)	363 (42.1)	287 (45.9)	0.152	234 (44.3)	243 (50.0)	0.070
Age, years	75.8 (10.2)	73.3 (9.6)	< 0.0001	75.4 (10.2)	74.2 (9.1)	0.066
Male Gender, n (%)	515 (59.7)	384 (61.3)	0.534	350 (66.3)	305 (62.8)	0.240
Duration of diabetes, years	5.5 (4.6)	6.4 (5.3)	0.001	6.1 (5.2)	5.2 (4.5)	0.004
Body mass index, kg/m^2^	31.9 (6.7)	32.5 (6.9)	0.090	31.9 (6.6)	32.1 (6.8)	0.647
Systolic blood pressure, mmHg	133 (13)	132 (13)	0.447	132 (13.1)	132 (12.4)	0.936
Total cholesterol, mmol/L	4.2 (1.1)	4.2 (1.0)	0.825	4.1 (1.0)	4.2 (1.0)	0.329
HbA1c, mmol/mol/%	54.7 (14.7) /7.2 (3.5)	58.0 (15.4)/7.5 (3.6)	0.702	57.3 (14.3)/7.4 (3.5)	56.8 (14.5)/7.3 (3.5)	0.606
Estimated glomerular filtration rate, mL/min/1.73m^2^	85 (82 to 90)	83 (78 to 90)	0.125	86 (83 to 90)	84 (79 to 90)	0.325
No of prior CVD or bleeding comorbidities^a^						
0	28 (3.3)	45 (7.2)	< 0.0001	19 (3.6)	34 (7.0)	0.824
1	135 (15.7)	102 (16.3)		92 (17.4)	79 (16.3)	
2	235 (27.3)	190 (30.4)		153 (29.0)	153 (31.5)	
3	237 (27.5)	146 (23.3)		150 (28.4)	131 (27.0)	
4	140 (16.2)	91 (14.5)		100 (18.9)	75 (15.4)	
5	64 (7.4)	35 (15.6)		11 (2.1)	6 (1.2)	
6	19 (2.2)	14 (2.2)		3 (0.6)	7 (1.4)	
7	3 (0.4)	3 (0.5)		0 (0.0)	1 (0.2)	
8	1 (0.1)	0 (0.0)		0 (0.0)	0 (0.0)	
No of prescriptions potentially correlating with bleeding/CVD^b^						
0	119 (13.8)	87 (13.9)	0.097	75 (14.2)	68 (14.0)	0.535
1	189 (21.9)	176 (28.1)		135 (25.6)	149 (30.7)	
2	163 (18.9)	132 (21.1)		103 (19.5)	108 (22.2)	
3	197 (22.9)	131 (20.9)		118 (22.4)	103 (21.2)	
4	148 (17.2)	78 (12.5)		88 (16.7)	52 (10.7)	
5	45 (5.2)	21 (3.4)		9 (1.7)	5 (1.0)	
6	1 (0.1)	1 (0.2)		0 (0.0)	1 (0.2)	
No of anti-diabetes agents/insulin^c^						
Diet	300 (34.8)	197 (31.5)	0.468	195 (36.9)	168 (34.6)	0.183
1	313 (36.3)	246 (29.3)		198 (37.5)	207 (42.6)	
2	164 (19.0)	114 (18.2)		101 (19.1)	89 (18.3)	
3	73 (8.5)	56 (9.0)		29 (5.5)	17 (3.5)	
4	12 (1.4)	12 (1.9)		5 (1.0)	5 (1.0)	
5	0 (0.0)	1 (0.2)		0 (0.0)	0 (0.0)	
No of anti-hypertensive agents^d^						
0	107 (12.4)	96 (15.3)	0.086	60 (11.4)	71 (14.6)	0.078
1	199 (23.1)	112 (17.9)		130 (24.6)	89 (18.3)	
2	248 (28.8)	201 (32.1)		154 (29.2)	164 (33.7)	
3	219 (25.4)	163 (26.0)		126 (23.9)	120 (24.7)	
4	75 (8.7)	47 (7.5)		50 (9.5)	36 (7.4)	
5	14 (1.6)	7 (1.1)		8 (1.5)	6 (1.2)	

CVD indicates cardiovascular diseases

Estimated glomerular filtration rate was presented as median (interquartile range)

^a^ Prior CVD or bleeding comorbidities include congestive cardiac failure, ischemic heart disease, stroke, valvular heart disease, venous thromboembolism, atrial fibrillation, hypertension, bleeding, and gastrointestinal bleeding

^b^ Prescriptions potentially correlating with bleeding/CVD include proton pump inhibitors for gastrointestinal disease, antiplatelet, antidepressant, corticosteroids, and statins

^c^ Anti-diabetes agents/insulin includes metformin, sulfonylurea, dipeptidyl peptidase-4 inhibitors, thiazolidinediones, sodium-glucose contransporter-2 inhibitors, glucagon-like peptide 1, and alpha glucosidase inhibitor

^d^ Anti-hypertensive treatment includes diuretics, alpha-blocker, calcium channel blocker, ARB/ACE

**Table 2:** The distribution of outcomes in the comparison cohorts

	Unmatched cohorts		Cohorts after coarsened exact matching	
	Incident NOAC users (N = 862)	Incident warfarin users (N = 626)	Incident NOAC users (N = 582)	Incident warfarin users (N = 486)
Hospitalisation mainly due to CVD, n (%)	190 (22.0)	63 (10.1)	91 (17.2)	44 (9.1)
Hospitalisation mainly due to bleeding, n (%)	50 (5.8)	33 (5.3)	25 (4.7)	18 (3.7)
Re-hospitalisation mainly due to CVD, n (%)	70 (8.1)	15 (2.4)	37 (7.0)	8 (1.7)

CVD indicates cardiovascular diseases

By coarsened exact matching, 528 incident NOAC users with type 2 diabetes were matched with 486 warfarin users with type 2 diabetes via 6 matching steps (Fig. 1). After coarsened exact matching, the matched variables tended to be closer (Tables 1, 3, Fig. 2). In particular, matched variables were very similar after samples were weighted by entropy matching (Fig. 2, Additional file 1: Table S1) in terms of mean, variance and skewness.

**Table 3:** Bleeding/CVD subtypes, anti-hypertensive treatments, and anti-diabetes treatments distribution in the unmatched and coarsened exact matched cohorts

	Unmatched cohorts		P-values	Cohorts after coarsened exact matching		P-value
	Incident NOAC users (N = 862)	Incident warfarin users (N = 626)		Incident NOAC users (N = 582)	Incident warfarin users (N = 486)	
Prior CVD or bleeding comorbidities						
Atrial fibrillation, n (%)	622 (72.2)	386 (61.7)	< 0.0001	383 (72.5)	349 (71.8)	0.530
Heart failure, n (%)	159 (18.5)	109 (17.4)	0.601	72 (13.6)	77 (15.8)	0.321
Cerebrovascular disease, n (%)	177 (20.5)	112 (17.9)	0.203	106 (20.1)	79 (16.3)	0.116
Hypertension, n (%)	584 (67.8)	409 (65.3)	0.956	337 (63.8)	311 (64.0)	0.329
Ischemic heart disease, n (%)	239 (27.7)	170 (27.2)	0.808	130 (24.6)	120 (24.7)	0.979
Valvular heart disease, n (%)	70 (8.1)	71 (11.3)	0.036	36 (6.8)	45 (9.3)	0.152
Venous thromboembolism, n (%)	78 (9.1)	68 (10.9)	0.246	39 (7.4)	46 (9.5)	0.233
Prior bleeding, n (%)	382 (44.3)	219 (35.0)	< 0.0001	202 (38.3)	163 (33.5)	0.118
Gastrointestinal bleeding, n (%)	28 (3.3)	20 (3.2)	0.954	16 (3.0)	10 (2.1)	0.328
Anti-hypertensive treatment						
Diuretics, n (%)	365 (42.3)	252 (40.3)	0.420	215 (40.7)	190 (39.1)	0.598
Beta-blocker, n (%)	450 (52.2)	338 (54.0)	0.495	280 (53.0)	267 (54.9)	0.543
Calcium channel blockers, n (%)	314 (36.4)	186 (29.7)	0.006	162 (30.7)	149 (30.7)	0.427
ARB/ACEi, n (%)	508 (58.9)	389 (62.1)	0.212	301 (57.0)	301 (61.9)	0.111
Anti-diabetes treatments						
Insulin, n (%)	122 (14.2)	97 (15.5)	0.471	63 (11.9)	64 (13.2)	0.552
Metformin, n (%)	445 (51.6)	329 (52.6)	0.722	264 (50.0)	238 (49.0)	0.743
Sulfonylurea, n (%)	171 (19.8)	133 (21.3)	0.506	92 (17.4)	84 (17.3)	0.953
Thiazolidinediones, n (%)	12 (1.4)	16 (2.6)	0.103	5 (1.0)	6 (1.2)	0.659
Dipeptidyl peptidase-4 inhibitors, n (%)	119 (13.8)	85 (13.6)	0.910	66 (12.5)	46 (9.5)	0.123
Sodium-glucose co-transporter-2 inhibitors, n (%)	24 (2.8)	23 (3.7)	0.333	10 (1.9)	13 (2.7)	0.404
Glucagon-like peptide 1, n (%)	12 (1.4)	12 (1.9)	0.428	6 (1.1)	5 (1.0)	0.869

**Fig. 3:**
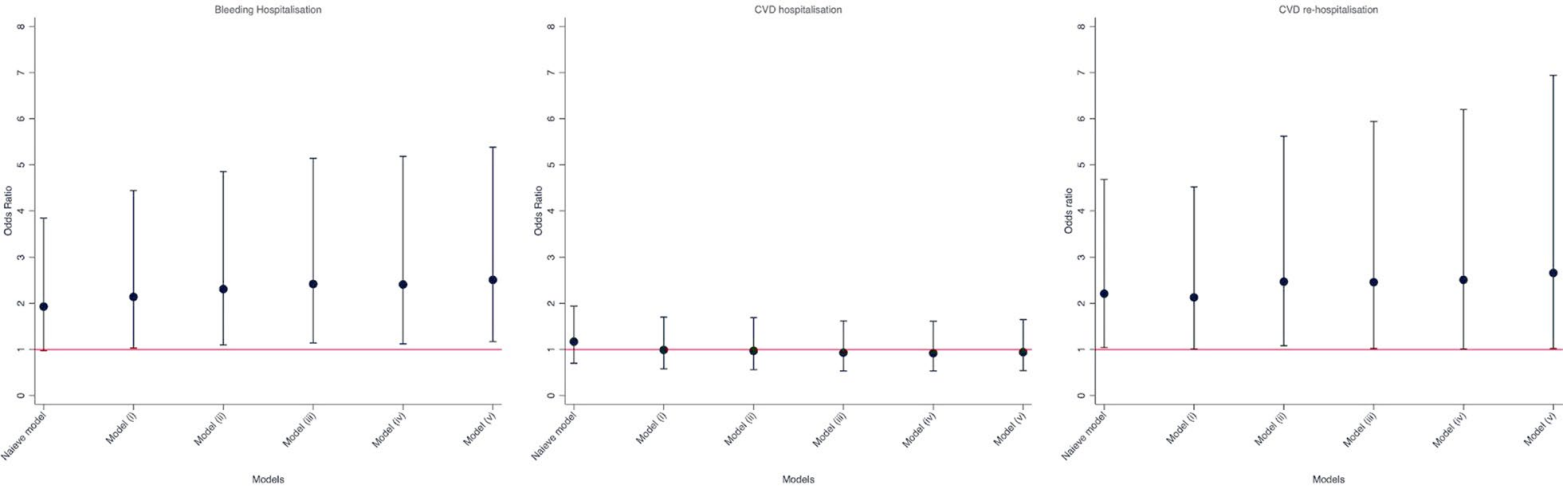
Adjusted odds ratios for association between NOAC (reference to warfarin) and risk of bleeding hospitalisation, CVD hospitalisation, CVD re-hospitalisation. Naive model weighted for age at incident anticoagulant prescription, gender, the CCG where their practices belong to, and duration of having recorded diagnosed type 2 diabetes by their incident anticoagulant prescription; model (i) weighted for all adjusted variables in model naïve model plus body mass index, systolic blood pressure, HbA1c and total cholesterol; model (ii) weighted for all adjusted variables in model (i) plus prior bleeding (gastrointestinal bleeding and other bleeding) and cardiovascular disease subtypes (hypertension, atrial fibrillation, heart failure, ischemic heart disease, cerebrovascular diseases, valvular heart disease, venous thrombosis); model (iii) weighted for all adjusted variables in model (ii) plus prescriptions potentially relevant to bleeding event (antidepressant, Statin, NSAIDS, Corticosteroid, proton pump inhibitor for gastrointestinal disease, and antiplatelet); model (iv) weighted for all adjusted variables in model (iii) plus anti-hypertensive treatment (diuretics, alpha-blocker, calcium channel blocker, ARB/ACE); model (v) weighted for all adjusted variables in model (iv) plus anti-diabetes treatments (insulin, metformin, sulfonylurea, thiazolidinediones, dipeptidyl peptidase-4 inhibitors, sodium-glucose co-transporter-2 inhibitors, glucagon-like peptide 1)

Compared with warfarin users, NOAC users were associated with increased risk of hospitalisation mainly due to bleeding (Fig. 3). The odds ratio weighted by matched variables in each matching step was 1.93 (95 confidence intervals 0.97–3.84) for model (i) weighted for age at incident anticoagulant prescription, gender, the CCG where their practices belonged, and duration of recorded diagnosed type 2 diabetes by their incident anticoagulant prescription; 2.14 (1.03–4.44) for model (ii) weighted for all adjusted variables in model (i) plus body mass index, systolic blood pressure, HbA1c and total cholesterol; 2.31 (1.10–4.85) for model (iii) weighted for all adjusted variables in model (ii) plus prior bleeding (gastrointestinal bleeding and other bleeding) and CVD subtypes (hypertension, atrial fibrillation, heart failure, ischemic heart disease, cerebrovascular diseases, valvular heart disease, venous thrombosis); 2.42 (1.14–5.14) for model (iv) weighted for all adjusted variables in model (iii) plus prescriptions potentially relevant to bleeding event (antidepressant, Statin, NSAIDS, Corticosteroid, proton pump inhibitor for gastrointestinal disease, and antiplatelet); 2.41 (1.12–5.18) for model (v) weighted for all adjusted variables in model (iv) plus anti-hypertensive treatment (diuretics, alpha-blocker, calcium channel blocker, ARB/ACE); 2.51 (1.17–5.38) for model (vi) weighted for all adjusted variables in model (v) plus anti-diabetes treatments (insulin, metformin, sulfonylurea, thiazolidinediones, dipeptidyl peptidase-4 inhibitors, sodium-glucose co-transporter-2 inhibitors, glucagon-like peptide 1).

Compared with warfarin users, NOAC users were not associated with a significant risk of hospitalisation due to CVD (Fig. 3). The weighted odds ratios were 1.17 (0.70–1.94), 0.99 (0.58–1.70), 0.97 (0.56–1.69), 0.93 (0.53–1.62), 0.92 (0.53–1.61) and 0.94 (0.54–1.65) for model (i) to model (vi), respectively.

However, compared with warfarin users, NOAC users were associated with a higher risk of re-hospitalisation due to CVD (Fig. 3). The weighted odds ratios were 2.21 (1.04–4.68), 2.13 (1.01–4.52), 2.47 (1.08–5.62), 2.46 (1.02–5.94), 2.51 (1.01–6.20), and 2.66 (1.02–6.94) for model (i) to model (vi), respectively.

## Discussion

### Main findings

Based on routinely collected primary care electronic health records linked with hospitalisation data in a population with type 2 diabetes, we have, for the first time, identified an increased risk of hospitalisation due to bleeding events and re-hospitalisation due to CVD associated with incident NOAC use compared with incident warfarin use. The risk of hospitalisation due to CVD did not significantly differ between incident NOAC and warfarin users.

### Strengths and limitations

A key strength of this work was the application of a novel, tapered matching method to form a ‘quasi-trial’ comparison sample to compare the risk of hospitalisation for the three outcomes between incident NOAC and warfarin users with type 2 diabetes. Through the tapered matching, we were able to transparently examine how differences in specific sets of confounders contributed to the risk of hospitalisation. By sequentially controlling for differences in demographic characteristics, clinical measurements, prior bleeding and CVD, prescriptions potentially relevant with bleeding, anti-hypertensive and anti-diabetes treatments, we observed how the risks of hospitalisation due to bleeding and CVD (re)hospitalisation compared after each match between new NOAC and the new warfarin users. This prospective cohort incorporating people with type 2 diabetes was derived from two independent primary care data bases in England that were linked with hospitalisation data. UK primary care electronic health records data (e.g. Clinical Practice Research Datalink) have been shown to be of good quality in terms of representativeness, coverage, validity, and consistency in records of comorbidities and prescriptions [20]. Hospitalisation data used in the study were complete as SUS data captures all hospitalisation information for patients and its recorded outcomes, which has also been proven to have good validity including those experiencing events outside of the CCG catchment [21]. There are some limitations in this study. First, as the sample size was restricted, instead of exactly matching each bleeding and CVD comorbidity, each anti-hypertensive prescription and each anti-diabetes prescription, we have matched the number of bleeding and CVD comorbidities, number of antihypertensive prescriptions and number of anti-diabetes prescriptions. However, we have compared the bleeding and CVD comorbidity, each antihypertensive prescription, and each anti-diabetes prescription after matching, no significant difference was identified between NOAC and warfarin user groups (Table 3). The risks of three outcomes were also examined by number of CVD or bleeding comorbidities, number of prescriptions potentially correlating with bleeding or CVD and number of anti-hypertensive agents. Neither in the NOAC or warfarin cohorts, did patients with more comorbidities, more prescriptions or more anti-hypertensive agents have statistically different risks of outcomes, compared with patients with less comorbidities, less prescriptions or less anti-hypertensive agents. Second, the outcomes were short-term (within 12 months since the first anticoagulant prescription). The long-term risks of these outcomes need to be examined in external longer-term studies. Third, death linkage was not accessible in this study, therefore the competing risk from death could not be evaluated. Fourth, due to the restrictions in sample size, the comparison between each NOAC with warfarin could not be made.

Some biomarkers (e.g. von Willebrand factor, fibrinogen and D-dimer) and prognostic factors of bleeding (Anti-IIa or anti-Xa levels) as potential matching variables were not available in the current study. Future replication studies with matching of these biomarkers are warranted.

Previous trials [22–29] and two meta-analysis [30, 31] revealed non-difference or benefits of NOAC for diabetes patients with AF. This differs from our target population which was the general type 2 diabetes population. Unfortunately, it was not possible for this study to apply a matching procedure in the subgroup of diabetes with AF due to its sample size. Future replication studies among diabetes patients with AF are warranted.

As the study was aimed to evaluate the impact of persistent exposure of each anticoagulant, a study of intermittent users after the first 30 days of use (for any reason e.g. adherence, side effect) has not been able to be included. Future studies addressing the dose–response relationship of time-varying exposure for each anticoagulant and outcomes are warranted.

Although some cases would have experienced switched anticoagulant prescriptions, the reasons for such a switch are not available and they might represent a specific anticoagulant user subgroup, The aim of the study was to compare the independent persistent exposure of NOAC and warfarin and in such a subgroup, a wash-out period between the two anticoagulants would be required, an unrealistic scenario in the routine primary care. Therefore, the risk from switching anticoagulant prescriptions should be evaluated in future studies.

INR information was not available in this study and INR monitoring and maintenance of a therapeutic/safe INR was seen as part of warfarin use (and one of the reasons for using NOACs).

### Comparison with prior studies

As NOACs do not require routine blood testing, they have been increasingly prescribed to replace the traditional anticoagulant, warfarin. Both observational studies and trials in the general population have suggested that NOACs are associated with less major bleeding events. Few studies, particularly cohort studies have explored differences between NOACs and warfarin among people with type 2 diabetes. In some trials, subgroup analyses have revealed heterogenous effects on bleeding among patients with diabetes. For example, in the ARISTOTLE trial [32] no difference in bleeding risk was shown between apixaban and warfarin in the group with diabetes. In ENGAGE AF-TIMI 48 trial bleeding was reduced in patients both with, and without, diabetes for edoxaban. Furthermore, in a meta-analysis of data from the pivotal NOAC trials [33], the risks of major bleeding with NOAC vs warfarin in patients with vs without diabetes were not statistically different (interaction, P = 0.12). Using our well-designed tapered matching method, we have formed quasi-trial comparison samples able to compare patients with type 2 diabetes using either NOAC or warfarin and revealed an increased risk of bleeding hospitalisation within 12 months after the initiation of NOAC comparing with warfarin. Moreover, we found there was no difference between NOAC and warfarin in terms of short-term risk of CVD hospitalisation; but increased risk of re-hospitalisation due to CVD within 12 months was observed in NOAC comparing with warfarin.

### Clinical implications

Findings in this study, if confirmed, suggests that caution may be required when prescribing the newer anticoagulants to patients with type 2 diabetes. This would mean continuation of the regular blood testing needed for warfarin but not NOACs. Different from the general population, the traditional anticoagulant warfarin might be safer than NOAC in terms of short-term risk of bleeding and CVD re-hospitalisation, both of which are associated with large health-costs [8]. Moreover, due the superiority of the tapered matching method, the impact of potential confounders on comparisons between NOAC and warfarin have been revealed. It has been found that among people with a prior history of bleeding or CVD, NOAC use would potentially increase the bleeding short-term risk of bleeding and CVD re-hospitalisation. Therefore, it might be wiser to prescribe warfarin for patients with type 2 diabetes and a prior history of bleeding or CVD. As we were limited by sample size, subgroups of patients with bleeding and those with prior CVD could not be analysed separately. Replication studies using other data are warranted.

Although a significant difference in risk of CVD hospitalisation was not identified in the current study, the significant risk of CVD re-hospitalisation was found in NOAC users, suggesting the association between NOAC and the risk of severe CVD condition as the severe CVD condition would be more likely to trigger CVD re-hospitalisation. Unfortunately, as a result of the limited sample size, the subtype of CVD events could not be further evaluated in the current study.

## Conclusion

Based on primary care data linked with hospitalisation data, via a novel tapered matching method, we formed ‘quasi-trial’ comparison cohorts for incident NOAC and warfarin users with type 2 diabetes. This study showed an increased short-term risk of major bleeding hospitalisation, and of CVD re-hospitalisation in new NOAC users comparing with new warfarin users. For patients with type 2 diabetes, caution is warranted when prescribing NOACs as the first anticoagulant treatment, particularly among patients with prior bleeding or a CVD history. Further large-scale replication studies in external datasets and replication studies with long-term outcomes and matching other possible confounders are warranted. Future comparative studies between subtypes of NOAC and warfarin, along with the development of specific risk algorithms for bleeding and CVD events in diabetes patients are also warranted.

## Supplementary information

**Additional file 1: Table S1.** Mean, variance and skewness between NOAC user group and warfarin user group after entropy matching.

## Data Availability

The detailed datasets analysed during the current study are available from Professor David Simmons on reasonable request.

## Abbreviations

NOACs: Non-vitamin K antagonist oral anticoagulants
CVD: Cardiovascular diseases
T2DM: Type 2 diabetes
CCGs: Clinical commissioning groups
SUS: Secondary User Service
