# Supplementary material for: Cardiovascular risks and bleeding with non-vitamin K antagonist oral anticoagulant versus warfarin in patients with type 2 diabetes: a tapered matching cohort study
Source: Cardiovasc Diabetol. 2020 Oct 10;19:174. doi: 10.1186/s12933-020-01152-y (PMC7548035; doi:10.1186/s12933-020-01152-y)
Supplement: Supplementary file 1 — Additional file 1: Table S1. Mean, variance and skewness between NOAC user group and warfarin user group after entropy matching. [file 12933_2020_1152_MOESM1_ESM.docx]

**Online-only Additional Material**

**Additional Table S1**. Mean, variance and skewness between NOAC user group and warfarin user group after entropy matching

*¶ Prior CVD or bleeding comorbidities include congestive cardiac failure, ischemic heart disease, stroke, valvular heart disease, venous thromboembolism, atrial fibrillation, hypertension, bleeding, and gastrointestinal bleeding.*

*§ Prescriptions potentially correlating with bleeding / CVD include proton pump inhibitors, antiplatelet, antidepressant, corticosteroids, and statins.*

¦ *Anti-diabetes agents / insulin includes metformin, sulfonylurea, dipeptidyl peptidase-4 inhibitors, thiazolidinediones, sodium-glucose contransporter-2 inhibitors, glucagon-like peptide 1, and alpha glucosidase inhibitor.*

** Anti-hypertensive treatment includes diuretics, alpha-blocker, calcium channel blocker, ARB/ACE.*

*CVD indicates cardiovascular diseases.*

|  | NOAC user group (n=528) | | | Warfarin user group (n=486) | | |
| --- | --- | --- | --- | --- | --- | --- |
|  | mean | variance | skewness | mean | variance | skewness |
| Male Gender | 0.7 | 0.2 | -0.7 | 0.7 | 0.2 | -0.7 |
| Age | 75.4 | 104.4 | -0.7 | 75.4 | 104.4 | -0.7 |
| Duration of diabetes | 6.1 | 27.4 | 0.8 | 6.1 | 27.4 | 0.8 |
| Clinical commission group - 2 | 0.4 | 0.2 | 0.2 | 0.4 | 0.2 | 0.2 |
| Body mass index | 31.9 | 43.8 | 0.9 | 31.9 | 43.8 | 0.9 |
| Systolic blood pressure | 132.1 | 172.5 | 0.4 | 132.1 | 172.5 | 0.4 |
| Total cholesterol | 4.1 | 1.0 | 1.2 | 4.1 | 1.0 | 1.2 |
| HbA1c | 57.2 | 200.0 | 1.5 | 57.2 | 200.0 | 1.5 |
| No of prior CVD or bleeding comorbidities ^¶^ | 2.5 | 1.4 | 0.0 | 2.5 | 1.4 | 0.0 |
| No of prescriptions potentially correlating with bleeding / CVD ^§^ | 2.1 | 1.9 | 0.1 | 2.1 | 1.9 | 0.1 |
| No of anti-diabetes agents / insulin ¦ | 1.0 | 0.9 | 0.8 | 1.0 | 0.9 | 0.8 |
| No of anti-hypertensive agents ^*^ | 2.0 | 1.5 | 0.1 | 2.0 | 1.5 | 0.1 |
